# How to analyze postoperative radiographs after total hip replacement

**DOI:** 10.1007/s11604-022-01332-8

**Published:** 2022-09-05

**Authors:** Martyna Barbara Budzińska, Bartosz Michał Maciąg, Krystian Żarnovsky, Tomasz Kordyaczny, Inga Magdalena Kowalczyk, Olga Adamska, Artur Stolarczyk

**Affiliations:** Department of Orthopaedics and Rehabilitation, Medical University of Warsaw, Międzyleski Specialist Hospital, 2 Bursztynowa Str., 04-749 Warsaw, Poland

**Keywords:** Total hip replacement, Radiological evaluation, Radiographs

## Abstract

Total hip replacement is one of the most widely performed surgeries. It is stated as the most efficient method of treating end-stage osteoarthritis of the hip joint. What is more, it significantly improves the quality of patients’ lives, relieves them from pain and restores decreased range of motion, provided that is conducted properly. Aim of this article is to indicate which constituents of prosthetic placement can be easily measured on postoperative radiographs and point out how to interpret obtained results. Multiple mechanical factors, such as center of rotation, femoral offset, acetabular offset, acetabular inclination, acetabular anteversion and leg length discrepancy can be measured on postoperative radiographs. To provide a successful surgery and to acquire both radiological and clinical satisfying results, proper prosthetic placement is crucial. Malpositioning of each element, in varying degrees may lead to dislocation or reoperation.

## Introduction

Total hip replacement (THR) is one of the most widely performed surgeries. There were 98.649 hip replacement procedures performed in 2019 in the United Kingdom [1]. Not only is it cost-effective treatment, but what is more important it is a very successful one, which relieves patients from pain, improves their quality of life and restores decreased range of motion. Unfortunately, there still exists a group of patients, which does not derive any advantage from THR. It is most often caused by the impact of mechanical aspects of THR on clinical outcome.

In everyday clinical practice, radiographs are the most widely used tool for imaging. It is the most widespread and commonplace method. Classical radiography is associated with a lower dose of radiation in comparison to computed tomography. Its advantage over other imaging methods results also from the lowest price. Computed tomography can be used for postoperative assessment as well. It is certainly more accurate than radiographs, but it has its limitations, such as radiation dose and limited availability [2]. There is also one method of imaging—magnetic resonance imaging, which is far less accessible and more expensive than previously mentioned imaging modalities. However, there are situations in which magnetic resonance imaging is helpful to obtain a diagnosis. They include tendinopathy, implants loosening, persistent postoperative pain or other conditions that are hard or even impossible to detect using computed tomography and classical radiography.

In connection with this, in the majority of cases, radiographs are perfectly adequate. What is more, the application of this imaging technique is not associated with the artifacts caused by metal implants, in contrast to the use computed tomography or magnetic resonance.

Aim of this article is to indicate which constituents can be measured on postoperative radiographs and point out how to interpret obtained results.

Multiple mechanical and biomechanical factors, such as center of rotation (COR), femoral offset, acetabular offset, acetabular inclination (AI), acetabular anteversion and leg length discrepancy can be measured on radiographs. Each one of them, in varying degrees, can affect the outcome of the surgery.

## Materials and methods

The literature was reviewed. Articles related to the subject, published in the years 1978–2021 were researched. Therefore publications including keywords such as total hip replacement, center of rotation, femoral offset, acetabular offset, cup anteversion, cup inclination and leg length discrepancy were searched in the PubMed database. Research was focused on English language papers, available abstracts, studies performed on people and articles. Inclusion of the articles was determined on the basis of titles, then abstracts, eventually entire articles. As terms of exclusion non-English language articles, papers ahead of print and only titles or abstract available were chosen. All studies presenting procedures conducted on animals were also excluded. If there occurred any signs of unreliability or relation to the topic were insignificant, the articles were eliminated during further evaluation. Afterwards, every selected article was verified another time. Any duplicates or obsolete information were removed. Research and error risk assessments were performed by one author. All information was selected and verified individually. Analysis and synthesis of studies were prepared independently.

All methods of measurements apply to radiographs taken in the supine position at a source-to-film distance of 100–115 cm with the X-ray beam directed to the midpoint of the pubic symphysis and perpendicular to the patient.

The described radiographic technique is a non-weight-bearing view. It is connected with the lesser radiation dose, allows to obtain a better quality radiographs, but it does not take into account the functional anatomy in contrast to weight-bearing view.

## Results

### Center of rotation (COR)

One of the goals of THR is to reconstruct the COR. As the study shows, restoring COR is an extremely important factor affecting operation result. It should be restored within 5 mm medial and 3 mm superior to the normal side. If optimal reconstruction is unattainable, the ability to control hip offset meticulously is limited. Shifting COR over 1 cm superiorly or medially causes early radiological signs of loosening. Malposition of COR may lead to abnormal gait, abductors insufficiency, increased risk of impingement and dislocation [3]. What is more, well reconstructed COR reduces the number of failures and revision surgeries [4]. Proper position of COR diminishes the risk of leg length discrepancy.

**Fig. 1:**
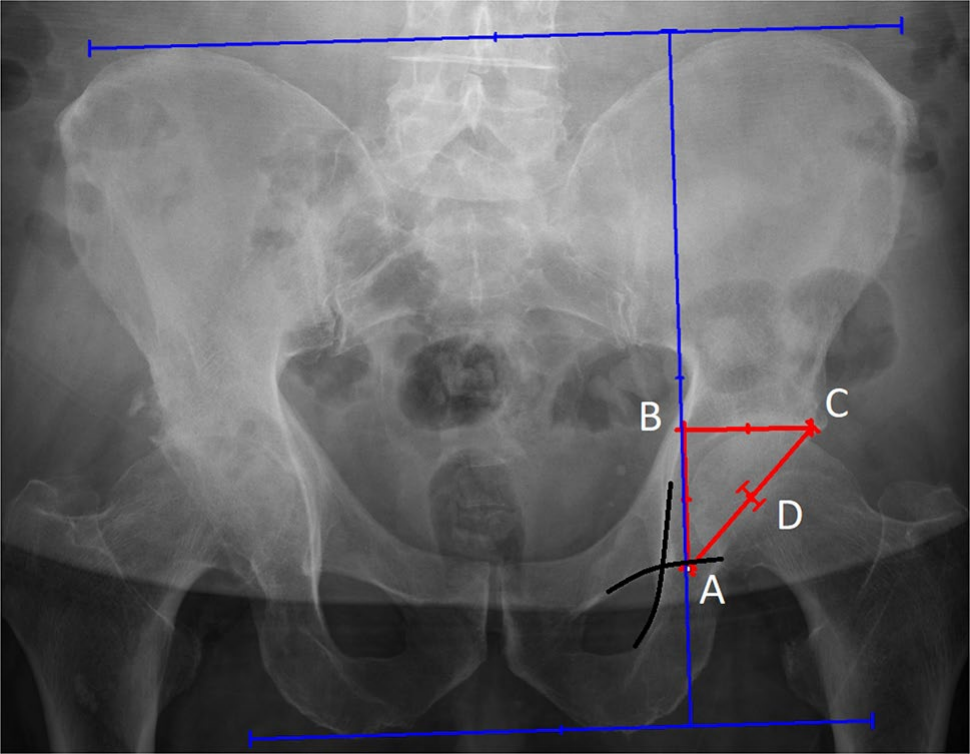
Determination of the COR. A. point located 5 mm lateral to the intersection of the Kohler’s and Shenton’s lines; B. point situated along the horizontal line at the level of the subchondral roof of the cup; at equal distance from both A and C points; C. point situated along the horizontal line at the level of the subchondral roof of the cup; D. point located half the length of the AC line

Ranawat’s method is an old but still applicable method used for the definition of the COR [5]. To determine the center of rotation on anteroposterior pelvis radiograph, two horizontal lines must be drawn. One at the level of iliac crests and the second one, at the level of ischial tuberosities. These lines must be connected by a perpendicular line passing through a point, which is located 5 mm lateral to the intersection of Kohler’s and Shenton’s lines. Point B and point C are situated along the horizontal line at the level of the subchondral roof of the cup. Point B is at an equal distance from both A and C points. COR is located half the length of the AC line [3, 5] (Fig. 1).

### Femoral offset

**Fig. 2:**
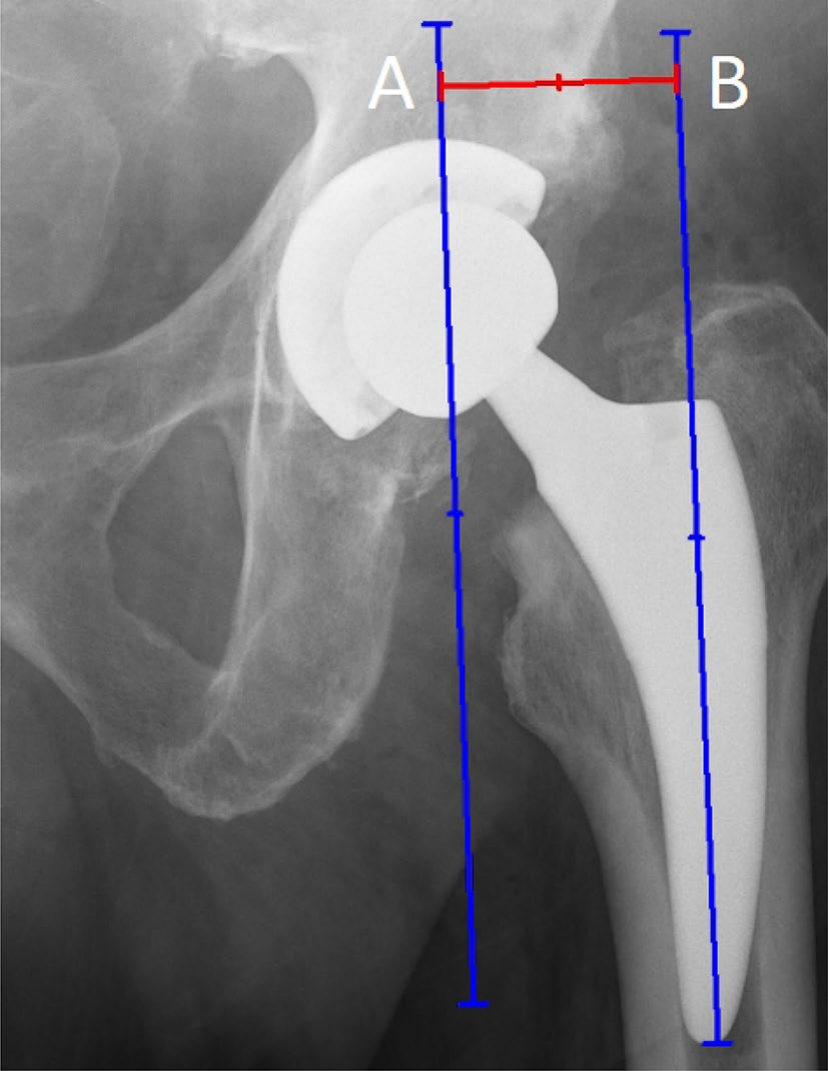
Femoral offset measurement. A. line passing through the COR; B. line bisecting the long axis of the femur

Femoral offset is stated as a distance from the COR of the femoral head to a line bisecting the long axis of the femur [3] (Fig. 2). Restored femoral offset enhances biomechanics, such as abduction strength and range of motion (ROM) by improving flexion and internal rotation of the hip [9, 10].

In addition, femoral offset restored within 5 mm to the normal side, decreases both linear and volumetric polyethylene wear [4]. Failing to restore offset < 5 mm may cause pain aggravation and deterioration of the joint. Reduced offset decreases soft-tissue tightness and predisposes to dislocation. According to the studies, well-positioned femoral offset may be the critical mechanical factor preventing dislocation after THR. However, it is still not stated which values of femoral offset are indications for revision [6–10].

### Acetabular offset

**Fig. 3:**
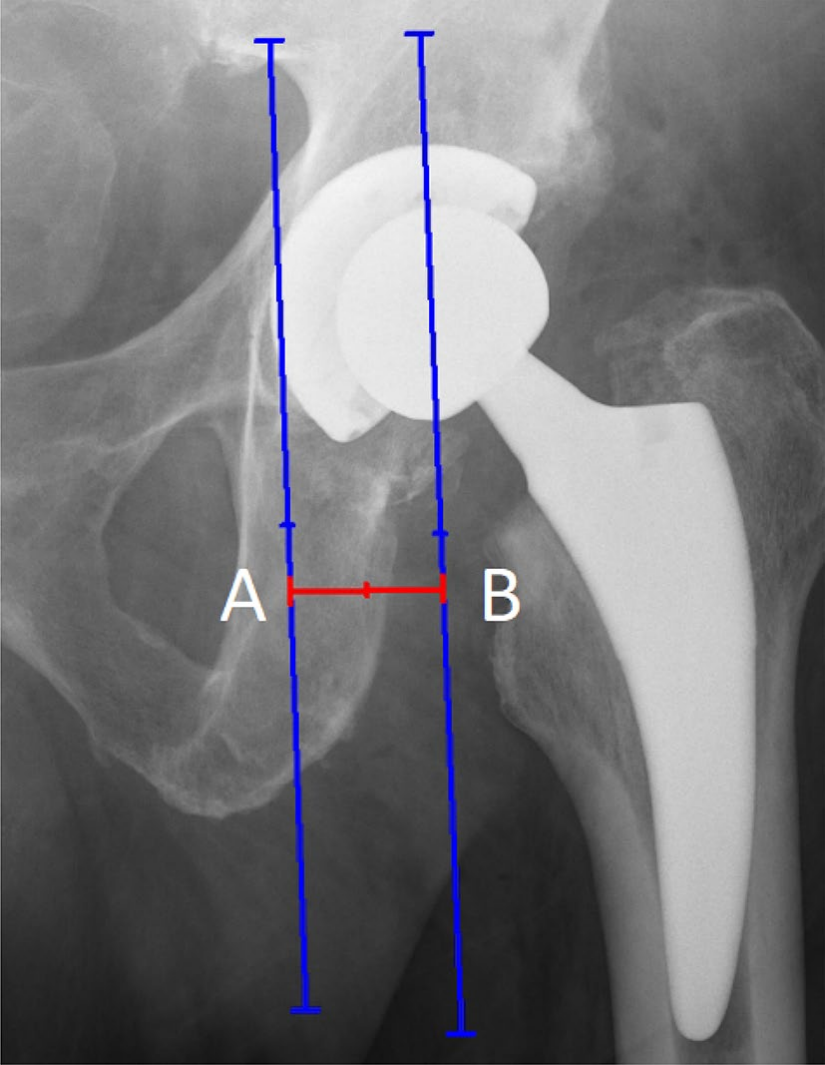
Acetabular offset measurement. A. line passing through the medial wall of the quadrilateral plate; B. line passing through the COR

Hip offset is not only femoral offset. There is another component part-acetabular offset, which should not be omitted because its value varies from person to person. Acetabular offset is the distance from COR to the medial wall of the quadrilateral plate [11, 12]. It is measured as a vertical distance between the center of rotation and teardrop on the same side (Fig. 3). The unreconstituted acetabular offset reduces the lever arm of body weight. As a result, gluteus medius and minimus muscles get a more vertical line of action [11].

### Acetabular inclination (AI)

**Fig. 4:**
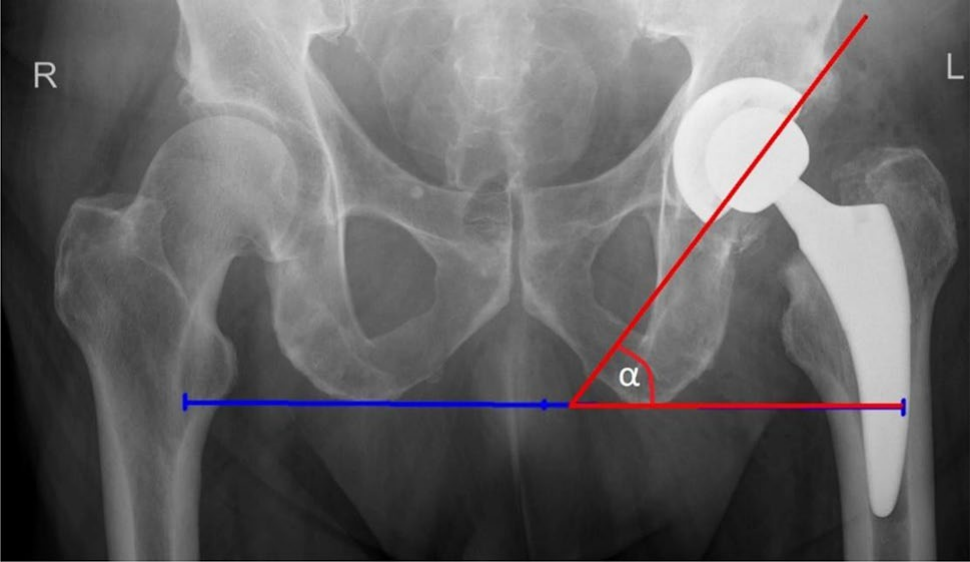
Acetabular inclination measurement. *α*—an angle between transischial line and a line conducted through the cup margin

Acetabular inclination is also known as an abduction angle. Radiographic AI is measured on anteroposterior radiographs. It is an angle between transischial line and a line conducted through the cup margins [10] (Fig. 4). AI affects range motion and wear of the acetabular component. When the abduction angle is lesser than 45°, flexion and abduction decreases. On the other hand, AI over 45° reduces adduction and rotation. There is also higher wear of acetabular polyethylene when the AI is over 45° [13, 14]. It cannot be univocally stated, without additional studies, what is a safe range for AI [15, 16].

### Acetabular anteversion

Computed tomography facilitates accurate determination of acetabular cup position, especially anteversion, but it is not widely used in clinical practice because of its high cost, limited availability and additional radiation exposure [17, 18]. Because of that, plain radiographs are in common use. Plenty of methods can be used to measure anteversion, and there is no validated and most efficient one [14]. This is due to the fact that pelvic tilt has a greater impact on measuring anteversion on anteroposterior radiographs rather than determining abduction angle on anteroposterior radiographs. Nevertheless, as studies show, there is a method that enables measurements that do not differ from measurements carried out using computed tomography. It is a method created by Liaw [19, 20].

Liaw’s anteversion is stated as:

$$ {\text{Anteversion}}\, = \,{\text{arc}}\,{\text{sic}}\, \, \left( {{\text{tan}}\,\alpha } \right) $$

**Fig. 5:**
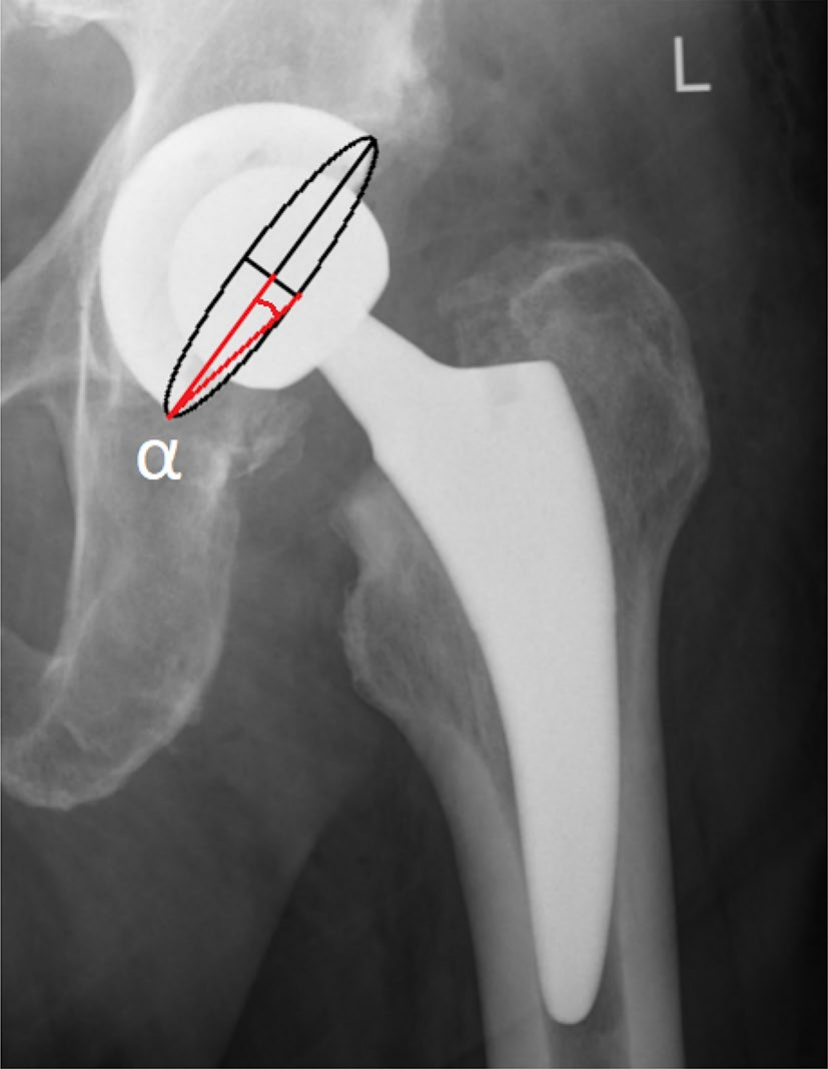
Liaw’s acetabular anteversion measurement. *α*—an angle between the principal axis of the ellipse and the vector connecting the endpoints of the main and the minor axes

*α* is an angle between the principal axis of the ellipse and the vector connecting the endpoints of the main and the minor axes. In plan, ellipse is located on the margin of a cup (Fig. 5).

The positioning of the cup influences the risk of dislocation. There is no consensus among researchers on what really is a safe zone for acetabular anteversion. According to Lewinnek safe zone theory, cup anteversion should be oriented between 5° and 25° to minimize dislocation after THR. But in fact, recent studies show that there is no real safe range, whether for AI or acetabular anteversion [14, 15, 21].

Anteversion values can be exploited to monitor acetabular migration. Change of anteversion on postoperative radiographs over 1.59° is an early sign of cup loosening, which can manifest itself in hip pain [18].

### Leg length discrepancy

Leg length discrepancy is one of the most frequent complications after THR. Lengthening occurs more often than shortening of the limb and is more noticeable in patients. According to the study, leg length discrepancy is perceived by patients when the operated limb is lengthened over 6 mm or shortened below 10 mm [22]. However, the greatest problem are inequalities above 10 mm due to their impact on everyday functioning. They can cause abnormal gait, instability, sciatica and back pain. Moreover, lengthening greater than 10 mm occurs with other complications: limping, pelvic obliquity and feeling disenchanted [15, 23].

**Fig. 6:**
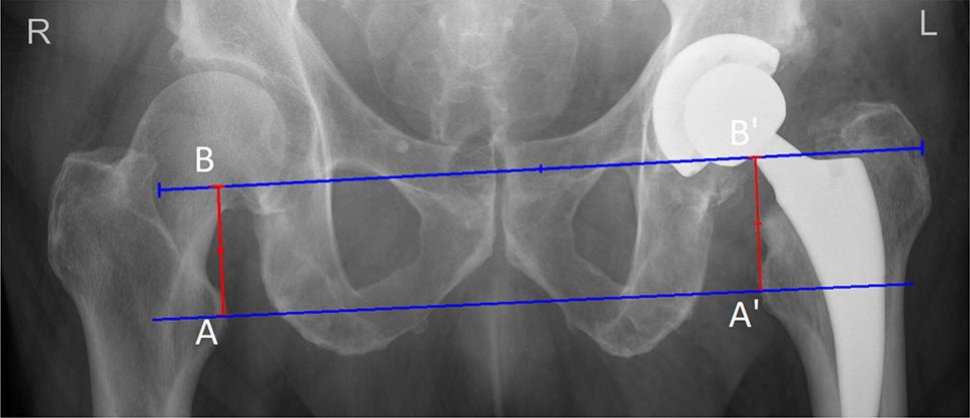
Leg length discrepancy measurement. A and A’ are located at the level of the tips of the lesser trochanters. B and B’ are situated at the level of the lower edge of the teardrop points. AB and A’B’ lines are distances between a line passing through the lower edge of the teardrop points to the corresponding tip of the lesser trochanter. Difference between AB and A’B’ lengths is stated as leg length discrepancy

The leg length discrepancy on anteroposterior radiographs is given as the difference in perpendicular distance between a line passing through the lower edge of the teardrop points to the corresponding tip of the lesser trochanter [10] (Fig. 6).

## Conclusions

To provide a successful surgery and to acquire both radiological and clinical satisfying results, all of the mechanics and biomechanics must be restored, if it is not, surgery can aggravate the patient’s complaints instead of removing them and the results of performed THR can be unsatisfactory. This may also result in dislocation or the need for reoperation.
